# Sampling Date, Leaf Age and Root Size: Implications for the Study of Plant C:N:P Stoichiometry

**DOI:** 10.1371/journal.pone.0060360

**Published:** 2013-04-02

**Authors:** Haiyang Zhang, Honghui Wu, Qiang Yu, Zhengwen Wang, Cunzheng Wei, Min Long, Jens Kattge, Melinda Smith, Xingguo Han

**Affiliations:** 1 State Key Laboratory of Forest and Soil Ecology, Institute of Applied Ecology, Chinese Academy of Sciences, Shenyang, China; 2 State Key Laboratory of Vegetation and Environmental Change, Institute of Botany, Chinese Academy of Sciences, Beijing, China; 3 Max Planck Institute for Biogeochemistry, Jena, Germany; 4 Department of Biology, Graduate Degree Program in Ecology, Colorado State University, Fort Collins, Colorado, United States of America; USDA-ARS, United States of America

## Abstract

Plant carbon : nitrogen : phosphorus (C:N:P) ratios are powerful indicators of diverse ecological processes. During plant development and growth, plant C:N:P stoichiometry responds to environmental conditions and physiological constraints. However, variations caused by effects of sampling (i.e. sampling date, leaf age and root size) often have been neglected in previous studies. We investigated the relative contributions of sampling date, leaf age, root size and species identity to stoichiometric flexibility in a field mesocosm study and a natural grassland in Inner Mongolia. We found that sampling date, leaf age, root size and species identity all significantly affected C:N:P stoichiometry both in the pot study as well as in the field. Overall, C:N and C:P ratios increased significantly over time and with increasing leaf age and root size, while the dynamics of N:P ratios depended on species identity. Our results suggest that attempts to synthesize C:N:P stoichiometry data across studies that span regional to global scales and include many species need to better account for temporal variation.

## Introduction

Ecological stoichiometry, which balances multiple elements and integrates different scales from individuals to ecosystems, has greatly advanced our understanding of ecological dynamics and processes [1], [2]. By studying ecological stoichiometry we can investigate energy flow and material cycling across diverse ecosystems [3]. Recent attention has been given to the strict homeostasis and relative plasticity of plant carbon (C), nitrogen (N), and phosphorus (P) ratios because of its importance for plant growth and adaption under climate change [4], [5]. Stoichiometric homeostasis, the degree to which an organism maintains its C:N:P ratios despite various elemental composition of resources, appears to be a potentially important mechanism responsible for the structure, functioning, and stability of grassland ecosystems [6]. Alternatively, stoichiometric flexibility, which reflects plant intrinsic physiological adjustment of C:N:P ratios could increase performance in response to environmental fluctuations [7]. Therefore, it is important to investigate the patterns of stoichiometric flexibility within and among plant species [5].

Variability in plant C:N:P stoichiometry across diverse habitats emerges from two interacting processes: 1) macro-scale constraints caused by specific geographic environment (i.e. climate and soil), and 2) fundamental physiological constraints resulting from plant growth, development, metabolism, phenological and life history traits [8]. N:P ratios in green foliage and live fine roots tend to be greatest near the equator and decline with latitude, indicating the impact of soil and climate on macro-scale stoichiometric flexibility [4], [9], while soil and climate impacted the variations of foliar C:N ratio by changing plant species composition [10]. On the other end of the spectrum, there is a “dilution” effect in N and P concentrations with the growth of plants [11]. Plant size, changing with seasonal development, has an influence on growth rate as indicated by metabolic scaling theory [12], [13] which in turn affects the stoichiometric ratios through metabolic changes [14]. Therefore, the C:N:P stoichiometric ratios can vary within species during plant ontogeny [15], [16]. Although plant nutrient status and its seasonal and ontogenetic variations have a long history of study within agricultural and plant ecophysiological fields [17]–[21], current ecological studies mainly focused on development stages (i.e. seeding, mature, fruiting, etc.). However, even within the same growth stage, sampling date and organ size may cause variation of plant stoichiometry [3]. Unfortunately, although sampling times can vary from months to years (or different years at a similar date), sampling date and organ size effects within a growth period (within a year of study) are often not held constant [4], [9]. Therefore, the extent to which sampling date and organ size affect plant C:N:P ratios when compared to species identity effects remains unknown.

Here we evaluated how sampling date, leaf age and root size within a growing season influence variation of plant C:N:P stoichiometry for grassland species in Inner Mongolia. Our objectives were to (1) examine how and to what extent the variation of plant C:N:P stoichiometry is affected by different sampling dates, age of leaves and size of roots, and (2) compare these effects with species identity effects on C:N:P stoichiometry.

## Materials and Methods

### Field Mesocosm Study

This study was conducted in 2006 at the Inner Mongolia Grassland Ecosystem Research Station (IMGERS, 43°26′N, 116°04′E, 1100 m a.s.l). Three species, representing the dominant and subdominant grasses and a minor annual forb in Inner Mongolian grasslands, were selected for the study: *Leymus chinensis* (a perennial C_3_ rhizomatous grass), *Cleistogenes squarrosa* (a perennial C_4_ bunchgrass) and *Chenopodium glaucum* (an annual C_3_ forb). To limit genetic variation, we collected seeds of each species within 1 m^2^ field plots in a grassland dominated by *L. chinensis* that had been fenced since 1999. The seeds were planted in replicate pots (30 cm diameter, 35 cm height) filled with sand on May 1, and the pots were placed in the field and covered when it rained. For additional details about the design, see [22]. Each pot had four holes at the bottom to allow for adequate drainage and received 250-mL solutions every day to prevent water limitation and to maintain a relatively constant macro- and micronutrient concentration. The macroelement composition of the solution was based on the formula developed by Hoagland & Arnon [23] and the microelement composition was followed Jensen & Collins [24]. There were a total of 36 pots for each species; three pots were randomly allocated to a replicate block and three replicate blocks were harvested on each sampling date (4 total). Upon seedling establishment, individuals in pots were thinned to 10–30 individuals, depending on plant size. We note that there was no shading effect in this experiment because the density was controlled to ensure that individuals within each pot did not shade each other.

To study the effects of sampling date, leaf age and root size, 30 individual plants of each species within the 3 pots of a replicate block were harvested at 15-day intervals from 10 July to 25 August, 2006 for a total of 4 sampling dates. From each individual plant we picked two healthy and fully expanded leaves from the mid-point of each plant. To study effect of leaf age, we also sampled young and old leaves on 25 August, 2006. For the young leaves, we sampled the newly-expanded leaves at the top of each plant. For the old leaves, we sampled the fully expanded leaves at the bottom of the stems of each plant [25]. Thus, there were three categories of leaf age evaluated: young, medium (leaves collected from mid-height), and old. Roots of each individual sampled were carefully washed in water and then separated based on size classes of root diameters (small<or  = 2 mm, medium between 2–5 mm, and large>or  = 5 mm) [9]. For each size class, we collected an average of 10 root segments of about 20.0 mm average length from each individual. Leaves and roots were oven-dried at 60°C, ground, and screened with 0.1-mm mesh for C, N and P content analysis.

### Natural Grassland Study

We collected samples in a *L. chinensis* dominated grassland located in Inner Mongolia, in which large mammal grazers had been excluded since 1999. The mean annual temperature of the study area is 0.3°C with mean monthly temperatures ranging from −21.6°C in January to 19.0°C in July. Long term (1980–2006) mean annual precipitation is 346 mm with a range from 166 mm in 2005 to 507 mm in 1998 [22]. During the study period, the annual precipitation was 304 mm in 2006 and 240 mm in 2007. Apart from precipitation, the availability of N rather than P limits ecosystem productivity in this region [6]. Aboveground plant tissue was sampled by clipping all plants at the soil surface within 6 1×1 m quadrats on 20 July and 20 August of 2006 and 2007, respectively. All living vascular plants were sorted to species and fifty healthy and fully expanded leaves of each of 13 species for each plot were collected. Tissue was oven-dried at 60°C, ground, and homogenized for C, N and P content analysis.

### Chemical Analysis

Total organic carbon concentration (% of dry mass) was determined using the method of Walkley & Black [26]. Briefly, 0.01 g dry samples were digested with 10 mL 0.50 mol·L^−1^ K_2_Cr_2_O_7_ at 180°C for 5 minutes followed by titration of the digests with standardized FeSO_4_. Total N concentration (% of dry mass) was analyzed by the Kjeldahl determination method [27], using H_2_SO_4_ and H_2_O_2_ for digestion, and NH_3_ was captured by H_3_BO_3_, then titrated by H_2_SO_4_. P content (% of dry mass) was measured using the same digestion solution for N followed by molybdenum stibium anti - mix reagent colorimetric analysis [28], standardized against known reference material.

### Data Analysis

Levene’s test was used to test for normality of all data before statistical analysis. For the field mesocosm study, multivariate ANOVA was used to test the effects of sampling date, leaf age, root size and species identity on plant C:N:P stoichiometry. For the natural grassland study, the same method was used to test for the effects of species, sampling month, sampling year and their possible interactions on plant C:N:P stoichiometry. Significant differences among treatment means were analyzed using Tukey’s multiple comparison post hoc tests. The total variance was partitioned into species, sampling date, leaf age, root size and residual components using the residual maximum likelihood (REML) method [29], [30]. All statistical analyses were performed on the R statistical platform [31].

## Results

### Mesocosm Study – Leaf Stoichiometry

**Figure 1:**
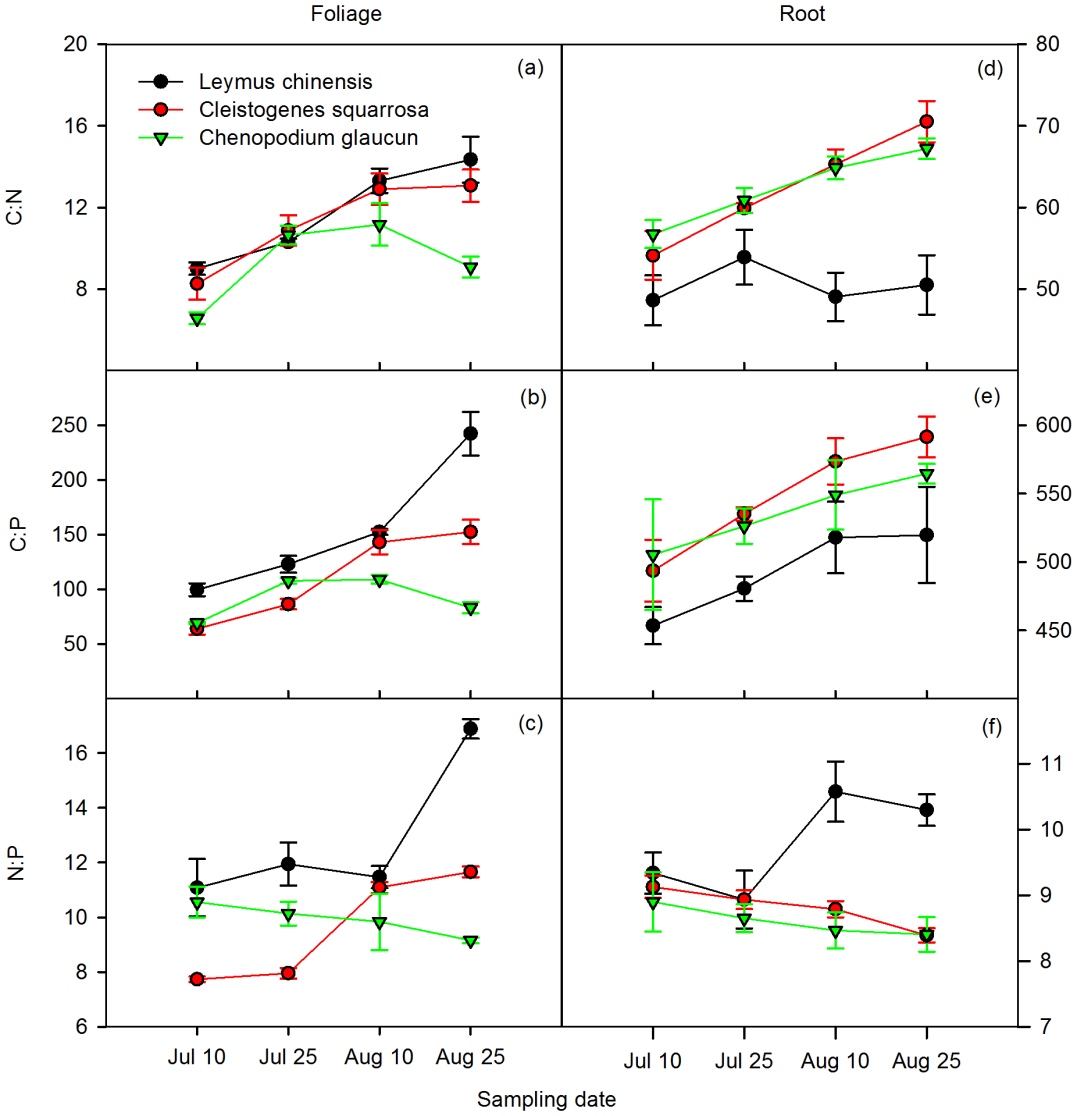
Change in C:N, C:P, N:P ratios for leaf (left) and root (right) tissue over time for three grassland species in the sand culture study. Error bars are SEM.

**Figure 2:**
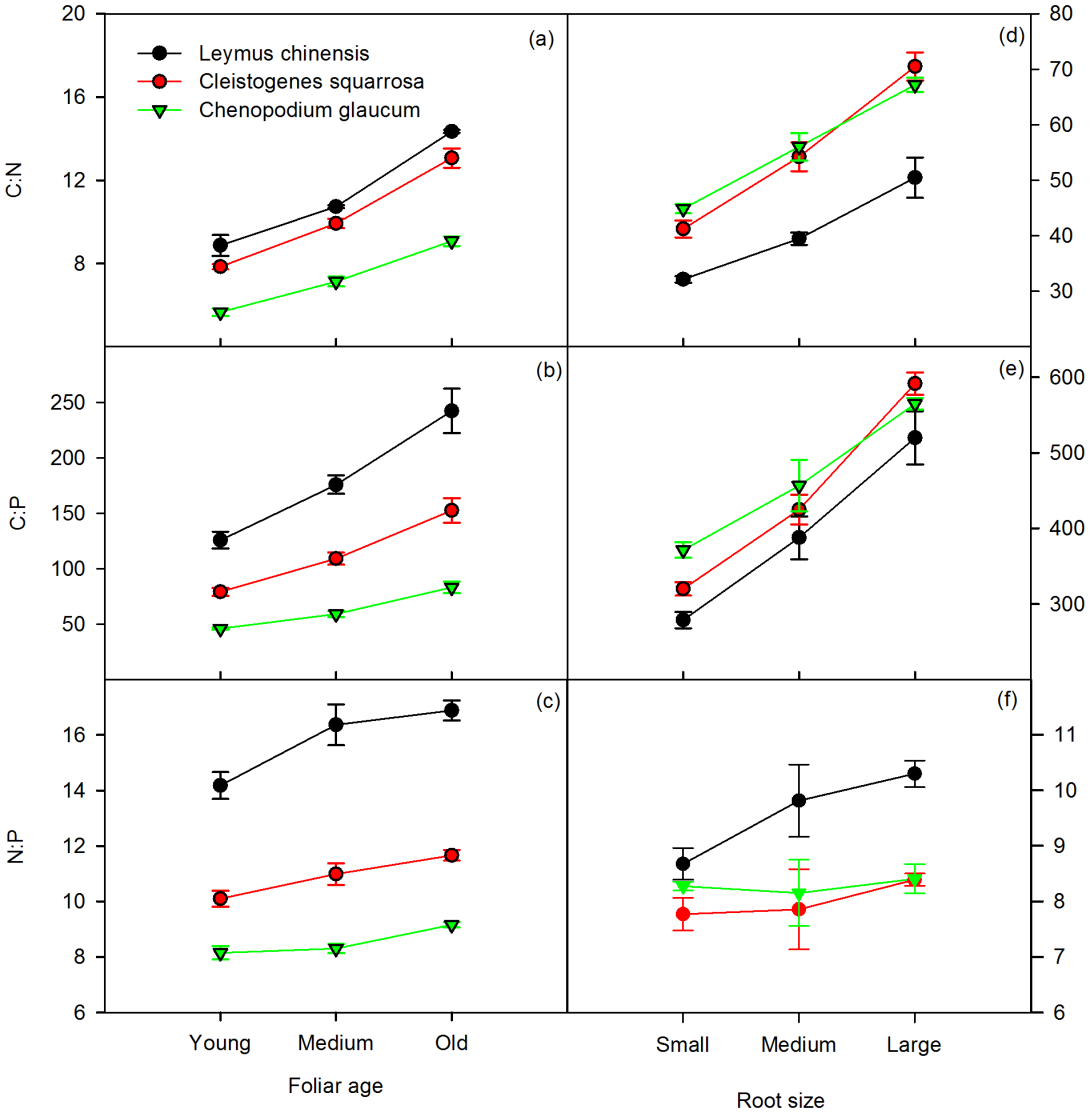
Mean C:N, C:P, N:P ratios for foliage of different ages (young = emerging; medium = fully expanded at midpoint of plant; old = fully expanded at base of plant) (left) and roots in different size categories (small = Φ ≤2 mm; medium = 2 mm <Φ<5 mm; large = Φ≥5 mm) (right). Error bars are SEM.

Species identity, sampling date, leaf age and their interactions (species identity x sampling date, species identity x leaf age) significantly affected C:N:P stoichiometry of leaves (*P*<0.001, Table 1). C:N, C:P and N:P ratios in leaf tissue increased over time, except for *C. glaucum* (Fig. 1a–c). For *L. chinensis*, C:N, C:P and N:P ratios were highest among the three species and significantly increased over time, except for N:P which did not significantly increase until the last sampling date (*P*<0.001, Fig. 1c ). However, for *C. glaucum*, C:N and C:P ratios increased for the first two sample dates, and then declined after August 10. The N:P ratio consistently decreased over time (Fig. 1c). C:N, C:P and N:P ratios all increased with leaf age (Fig. 2). For *L. chinensis*, C:N, C:P, and N:P ratios were the highest among the three species, and C:N, C:P ratios significantly increased with leaf age (*P*<0.01, Fig. 2). Similar patterns were observed for *C. squarrosa* and *C. glaucum*, while the latter had the lowest values (Fig. 2). As a whole, apart from a strong species × sampling date interaction on C:P ratios, sampling date had a stronger effect than species identity on foliar C:N and C:P ratios, while species identity had a stronger effect on the N:P ratio (Table 2). Similarly, leaf age explained a greater proportion of the variation for C:N than species identity, but in the case of C:P and N:P ratios, species identity explained a greater amount of variation than leaf age (Table 2). Species identity, leaf age, and their interaction significantly affected leaf C:N:P stoichiometry (*P*<0.001,Table 1).

**Table 1:** Multivariate ANOVA results for the effects of species, plant sampling date, leaf age and root size on C:N, C:P, N:P ratios in the sand culture study.

Factors		Leaf			Root		
		C:N	C:P	N:P	C:N	C:P	N:P
Sampling date(SD)	Species (df = 2)	F = 39.60	F = 171.56	F = 122.69	F = 96.31	F = 21.43	F = 55.87
		*P*<0.001	*P*<0.001	*P*<0.001	*P*<0.001	*P*<0.001	*P*<0.001
	SD (df = 3)	F = 79.99	F = 160.56	F = 46.01	F = 24.44	F = 21.33	F = 3.48
		*P*<0.001	*P*<0.001	*P*<0.001	*P*<0.001	*P*<0.001	*P = *0.031
	Species×SD (df = 6)	F = 8.62	F = 50.51	F = 34.65	F = 7.24	F = 0.59	F = 11.43
		*P*<0.001	*P*<0.001	*P*<0.001	*P*<0.001	*P = *0.739	*P*<0.001
Age or size(AS)	Species (df = 2)	F = 118.30	F = 404.47	F = 1152.33	F = 139.44	F = 24.89	F = 21.00
		*P*<0.001	*P*<0.001	*P*<0.001	*P*<0.001	*P*<0.001	*P*<0.001
	AS (df = 2)	F = 127.30	F = 166.46	F = 64.33	F = 250.37	F = 273.35	F = 3.00
		*P*<0.001	*P*<0.001	*P*<0.001	*P*<0.001	*P*<0.001	*P = *0.075
	Species×AS(df = 4)	F = 4.00	F = 15.12	F = 7.33	F = 4.77	F = 2.81	F = 2.00
		*P*<0.05	*P*<0.001	*P*<0.001	*P*<0.01	*P = *0.057	*P = *0.138

**Table 2:** Partitioning of total variation (%) of C:N, C:P, N:P into species, effects of sampling (sampling date, leaf age, root size) and residual components in the sand culture experiment.

Variation source	Plant sampling date								Organ age or size							
	Leaf				Root				Leaf age				Root size			
	C:N	C:P	N:P		C:N	C:P	N:P		C:N	C:P	N:P		C:N	C:P	N:P	
Species	17.84	0.00	12.16		59.91	34.92	27.12		44.26	65.21	93.49		35.19	7.96	59.52	
Effects of sampling	59.56	5.48	0.00		15.54	46.34	0.00		48.89	26.22	4.43		60.85	87.87	4.88	
Species×Effects of sampling	14.39	90.25	81.28		17.17	0.00	60.18		3.16	7.08	1.18		0.53	0.89	6.80	
Residual	8.21	4.27	6.56		7.37	18.74	12.70		3.69	1.48	0.90		3.43	3.27	28.79	

### Mesocosm Study – Root Stoichiometry

Species identity, sampling date and root size significantly affected C:N:P stoichiometry of roots (*P*<0.001,Table 1). For *C. squarrosa* and *C. glaucum* root C:N and C:P ratios increased with plant sampling date, while the N:P ratio decreased (Fig. 1f). *L. chinensis*, showed no significant differences between the sampling dates for root C:N, while C:P and N:P ratios of roots significantly increased with sampling date (*P*<0.01, Fig. 1). In addition, root size significantly affected C:N and C:P ratios of roots (*P*<0.001,Table 1), with both increasing with increasing root age (Fig 2d–f). There also was significant interaction between species identity and root size on root C:N ratios. Although root C:N increased for all three species, C:N ratios were consistently lower for *C. glaucum* (Fig. 2). Overall, sampling date had a stronger effect (i.e., explained a greater percentage of variation) than species identity on C:P ratio of roots, whereas species identity had a stronger effect on C:N and N:P ratios (Table 2). In contrast, the fraction of variation for C:N and C:P ratios explained by root size was greater than that explained by species identity, but it was lower for N:P ratios (Table 2).

### Natural Grassland Study – Leaf Stoichiometry

**Figure 3:**
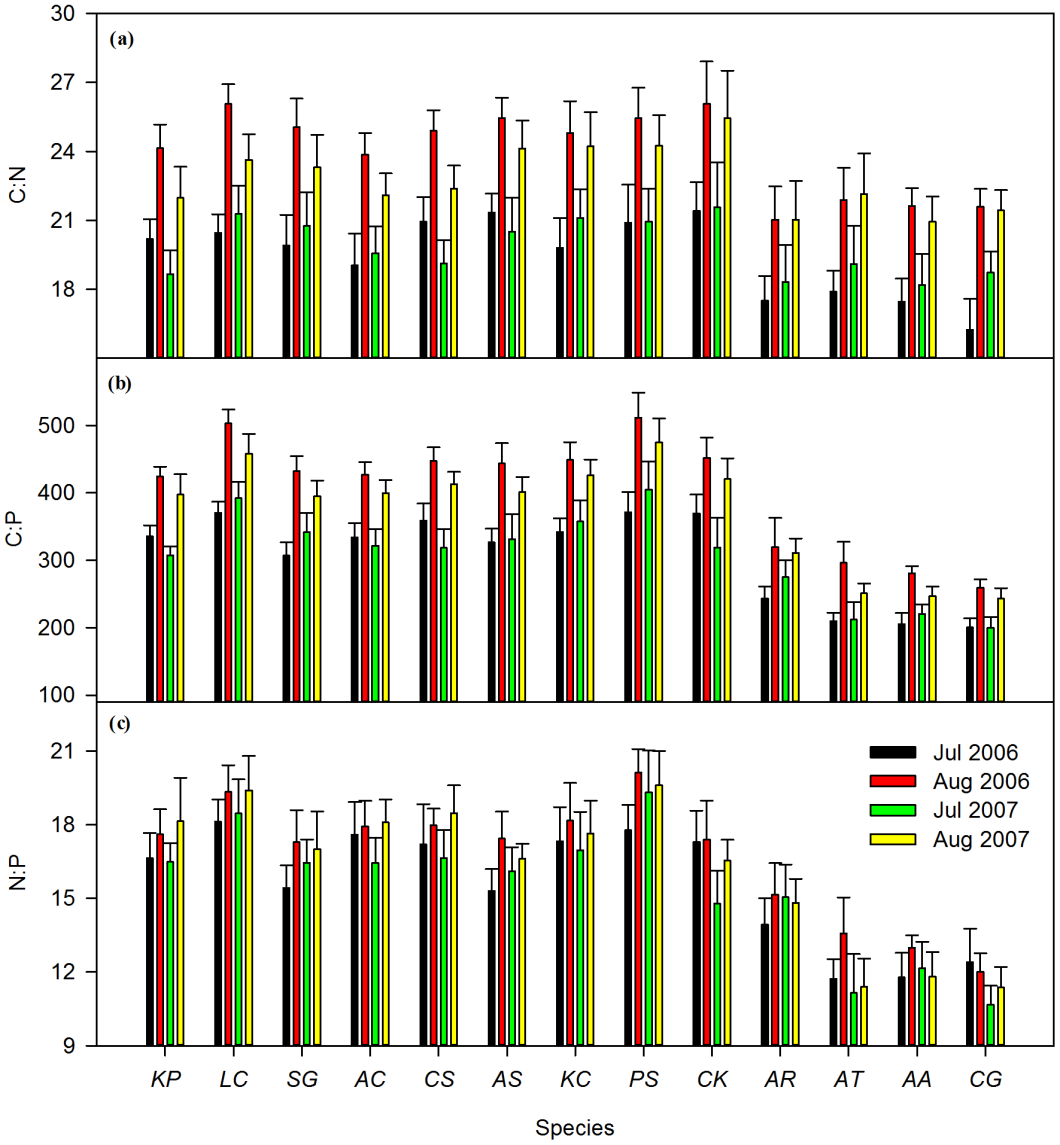
C:N, C:P, N:P ratios of 13 Inner Mongolia grassland dominate and common species with four sampling dates during two-year field N and P addition experiment. *KP Kochia Prostrate, LC Leymus chinensis*, *SG Stipa grandis*, *AC Agropyron cristatum*, *CS Cleistogenes squarrosa*, *AS Achnatherum sibiricum*, *KC Koeleria cristata*, *PS Poa sphondylodes*, *CK Carex korshinskyi*, *AR Allium ramosum*, *AT Allium tenuissimum*, *AA Axyris amarantoides*, *CG Chenopodium glaucum*. Error bars are SEM.

Both species identity and sampling month exhibited significant effects on leaf C:N, C:P and N:P ratios, while sampling year significantly affected only C:N and C:P ratios (Table 3). C:N, C:P and N:P ratios for leaves sampled in August were significantly higher than those of leaves sampled in July (*P*<0.001, Fig. 3). The foliar C:N, C:P and N:P ratios of the three local species from field mesocosm study were similar from the natural grassland study; that is, *L. chinensis*>*C. squarrosa*>*C. glaucum*. Overall, sampling month determined the greatest amount of variation of leaf C:N ratios, species identity determined the greatest amount of variation of N:P ratios, while C:P ratios were influenced by both species identity and sampling month (Table 4).

**Table 3:** Multivariate ANOVA results for the effects of species, sampling year (Y) and sampling month (M) on leaf C:N, C:P, N:P ratios in the field study.

Ratios	Species	Y	M	Species×Y	Species×M	Y×M	Species×Y×M
	(df = 12)	(df = 1)	(df = 1)	(df = 12)	(df = 12)	(df = 1)	(df = 12)
C:N	F = 112.98	F = 7.63	F = 717.29	F = 25.31	F = 0.07	F = 29.11	F = 0.28
	*P*<0.001	*P*<0.01	*P*<0.001	*P*<0.001	*P = *0.794	*P*<0.001	*P = *0.594
C:P	F = 524.53	F = 10.91	F = 330.48	F = 0.51	F = 9.05	F = 14.16	F = 0.002
	*P*<0.001	*P = *0.001	*P*<0.001	*P = *0.477	*P*<0.01	*P*<0.001	*P = *0.966
N:P	F = 569.98	F = 3.59	F = 35.23	F = 5.56	F = 2.81	F = 1.03	F = 0.65
	*P*<0.001	*P = *0.058	*P*<0.001	*P = *0.019	*P = *0.094	*P = *0.311	*P = *0.421

**Table 4:** Partitioning of total variation (%) of C:N, C:P, N:P into species, sampling year (Y), sampling month (M) and residual components in the field experiment.

Variation source	Species	Y	M	Species×Y	Species×M	Y×M	Species×Y×M	Residual
C:N	15.81	0.75	64.40	3.10	0.00	2.69	0.11	13.14
C:P	48.97	0.66	39.06	0.49	9.69	2.10	1.05	5.99
N:P	74.28	0.05	5.55	1.93	0.00	0.08	2.02	16.09

## Discussion

### Sampling Date, Leaf Age and Root Size Determines the Dynamics of C:N and C:P Ratios

Species identity and effects of sampling - sampling date (even within the same growth stage or growing season), leaf age and/or root size - significantly influenced leaf and root stoichiometry of plants in our mesocosm study and in natural grassland. Plant C:N, C:P and N:P all increased significantly with sampling date, leaf age and root size, consistent with previous studies [9], [21]. However, sampling date (within season or between years), leaf age and root size explained the greatest amount of variation in leaf and root C:N and C:P ratios, while species identity primarily mediated variation in N:P ratios of leaves and roots. Thus, our study suggests that sampling leaves and roots at different time points can strongly influence plant stoichiometric ratios, particularly C:N and C:P ratios of leaves and roots, as a consequence of the phenology (ontogeny) of the plant, age of leaves or size of roots.

In general, both C:N and C:P ratios of leaves and roots increased with the increasing of sampling date within a growing season in this study. The increase in leaf and root C:N and C:P ratios with sampling date was most likely driven by increasing plant size (and thus C content), which leads to a “dilution” of N and P content over time [32], [33]. Young plants assimilate and grow simultaneously, so the demand for nutrients is relatively large because these elements are essential for plant growth and play key role in enzyme production [18]. As plants get older, structural material enriched in C accumulates, leading to higher C:N and C:P ratios [18], which may coincide with changing metabolic activity and/or different investment during ontogeny [14], [34]. Thus, over time C:N and C:P ratios may increase due to reduced allocation of nutrients to older leaves and to the dilution of nutrients overall as leaf area and root systems increase in size over time.

Variation in plant C:N and C:P ratios with leaf age appeared to correspond with patterns of plant ontogeny. We were able to separate ontogenetic effects from the effects of time *per se*, as we compared young and old leaves for a particular time point. Growth is most active in meristems (e.g. young leaves, shoot tips or inflorescences) while older leaves no longer increase in size, even though baseline physiological processes are maintained [33], [34]. Thus, as leaves increase in size and/or age, the increased leaf C:N and C:P ratios reflects the accumulation of compounds with higher carbon to nutrient ratios [9]. These results also may be caused by an increase in defensive substances, or lower nutrient concentrations due to retranslocation from old leaves to young ones more active in growth [34], [35]. At the same time, old leaves have a lower photosynthetic capacity than younger leaves because of self-shading, and thus plants shift allocation of nutrients to younger leaves to maximize C gain [11], [36]. However, for our study, there was almost no shading effect because there is plenty of space among plants, and thus mechanisms other than shading alone were responsible for the increase in C:N and C:P ratios with leaf age. Variation in root C:N and C:P ratios with root size had a similar pattern with leaf age. In this case, distinct functions (i.e., water and nutrition uptake with fine roots, transport and maintenance with coarse roots) may have resulted in variation of C:N and C:P ratios for different root sizes [9].

Here we documented that sampling date, leaf age, and root size explained more variation in C:N and C:P ratios than species identity. However, the effects of sampling are not controlled in most large scale studies. The mean coefficient of variation (CV) of leaf C:N among different sampling dates and leaf ages was 0.23, while CV of leaf C:N across Chinese grassland biomes was 0.32 [10]. The mean CV of root C:N and C:P among different sampling dates and root size was 0.15 and 0.17, while CV of leaf and root C:P on global scale was 1.36 and 1.50 respectively [9]. Although there are limitations to compare CVs from different sample sizes, our results suggest that if effects of sampling were considered in C:N:P stoichiometry studies at large spatial scales, that variation could be reduced greatly and the accuracy could be improved significantly.

### Species Identity Determines N:P Ratios

Effects of sampling explained a limited amount of variation in leaf and root N:P ratios, and in some cases none of the variation (Table 2 and 4). For the most part, species identity explained a majority of the observed variance of leaf and root N:P ratios. Our results therefore indicated that species identity rather than effects of sampling mediated the variability of N:P stoichiometry at the local scale (i.e. the same environmental background) for grassland species. This finding was consistent with previous studies of vascular plants at larger spatial scales, which showed that species identity and plant classifications explain a large fraction of the observed variation of N:P ratios [37]–[40]. However, in these cases N and P availability varied with temperature and soil, and thus confounded the effects of species identity [41]. Therefore, it remains unclear to what extent effects of sampling, species identity or environmental constraints determine observed N:P ratios. Our results showed that effects of sampling only weakly impacted the variation of leaf and root N:P ratios, however, this does not mean that effects of sampling should be ignored given that factors such as sampling date, leaf age and root size can affect N:P ratios in specific cases or for particular species (i.e., there were strong interactive effects between species identity and effects of sampling).

### Implications for Future Studies on Plant C:N:P Stoichiometry

Plant C:N:P stoichiometric flexibility has been intensively studied and shows substantial variation [4], [7], [8], [37], which in general is attributed to environmental constraints and/or species identity. Our study indicated that C:N and C:P ratios were strongly affected by sampling date, leaf age and root size, and therefore also should be taken into account when compiling datasets across large spatial scales (i.e., global pattern analysis). Despite the importance of effects of sampling to C:N:P stoichiometry, information about sampling date, plant and organ developmental state, sampling site of the studied plants, root size, etc. is seldom explicitly provided along with plant trait data [42]. We suggest that protocols for plant trait measurements should include such information. In order to address the variation caused by different plant developmental stages and organ ages, we suggest that additional information about measurement date, plant seasonal developmental and organ age or size should become part of standard measurement protocols of ecological plant stoichiometry.

Trait-based studies have become extremely useful in community assembly ecology [43], [44]. However, most approaches evaluate species traits by mean trait values neglecting variations caused by different individuals, sampling date, organ age and size. Our results suggest that the conflicting patterns of C:N:P stoichiometry across the world could be due to this unaccounted variation. Numerous recent studies have suggested that intra-specific trait variability significantly affects various ecological processes [45], [46]. Young and old organs within an individual plant might function differently. Instead of sampling only mature leaves, more advanced interpretations may require a more diverse sampling scheme rather than averaging over tissues (leaf and roots) of different sizes and ages [7].

Ågren & Weih [7] propose that stoichiometric variation within individuals (between organs of different ages) or between individuals of different sizes cannot be ignored when considering stoichiometry as a functional trait to describe community structure [43], [46]. Our study also suggests that caution should be used when considering C:N and C:P ratios as functional traits because seasonal development and organ age or size strongly mediate C:N and C:P ratios in leaf and root tissues. In contrast, our study shows that N:P ratios, which are determined mainly by species identity, could be used as a novel functional trait to understand plant growth, competition and species coexistence in plant communities [7], [47].

## References

[1] Elser JJ, Sterner RW, Gorokhova E, Fagan WF, Markow TA, et al (2000) Biological stoichiometry from genes to ecosystems. Ecol Lett 3: 540–550.

[2] Sterner R.W, Elser JJ (2002) Ecological stoichiometry: the biology of elements from molecules to the biosphere.Princeton:Princeton University Press. 584 p.

[3] Elser JJ, Dobberfuhl DR, MacKay NA, Schampel JH (1996) Organism size, life history, and N:P stoichiometry. Bioscience 46: 674–684.

[4] Reich PB, Oleksyn J (2004) Global patterns of plant leaf N and P in relation to temperature and latitude. Proc Natl Acad Sci U S A 101: 11001–11006. 15213326 10.1073/pnas.0403588101 PMC503733

[5] Elser JJ, Fagan WF, Kerkhoff AJ, Swenson NG, Enquist BJ (2010) Biological stoichiometry of plant production: metabolism, scaling and ecological response to global change. New Phytol 186: 593–608. 20298486 10.1111/j.1469-8137.2010.03214.x

[6] Yu Q, Chen QS, Elser JJ, He NP, Wu HH, et al (2010) Linking stoichiometric homoeostasis with ecosystem structure, functioning and stability. Ecol Lett 13: 1390–1399. 20849443 10.1111/j.1461-0248.2010.01532.x

[7] Ågren GI, Weih M (2012) Plant stoichiometry at different scales: element concentration patterns reflect environment more than genotype. New Phytol 194: 944–952. 22471439 10.1111/j.1469-8137.2012.04114.x

[8] McGroddy ME, Daufresne T, Hedin LO (2004) Scaling of C : N : P stoichiometry in forests worldwide: Implications of terrestrial redfield-type ratios. Ecology 85: 2390–2401.

[9] Yuan ZY, Chen HYH, Reich PB (2011) Global-scale latitudinal patterns of plant fine-root nitrogen and phosphorus. Nat Commun 2: 344. 21673665 10.1038/ncomms1346

[10] He JS, Fang JY, Wang ZH, Guo DL, Flynn DFB, et al (2006) Stoichiometry and large-scale patterns of leaf carbon and nitrogen in the grassland biomes of China. Oecologia 149: 115–122. 16639565 10.1007/s00442-006-0425-0

[11] Reich PB, Ellsworth DS, Uhl C (1995) Leaf carbon and nutrient assimilation and conservation in species of differing successional status in an oligotrophic Amazonian forest. Funct Ecol 9: 65–76.

[12] Enquist BJ, Kerkhoff AJ, Huxman TE, Economo EP (2007) Adaptive differences in plant physiology and ecosystem paradoxes: insights from metabolic scaling theory. Glob Chang Biol 13: 591–609.

[13] Beardall J, Allen D, Bragg J, Finkel ZV, Flynn KJ, et al (2009) Allometry and stoichiometry of unicellular, colonial and multicellular phytoplankton. New Phytol 181: 295–309. 19121029 10.1111/j.1469-8137.2008.02660.x

[14] Rivas-Ubach A, Sardans J, Pérez-Trujillo M, Estiarte M, Peñuelas J (2012) Strong relationship between elemental stoichiometry and metabolome in plants. Proc Natl Acad Sci U S A 109: 4181–4186. 22371578 10.1073/pnas.1116092109 PMC3306711

[15] Méndez M, Karlsson PS (2005) Nutrient stoichiometry in Pinguicula vulgaris: nutrient availability, plant size, and reproductive status. Ecology 86: 982–991.

[16] Frost PC, Evans-White MA, Finkel ZV, Jensen TC, Matzek V (2005) Are you what you eat? Physiological constraints on organismal stoichiometry in an elementally imbalanced world. Oikos 109: 18–28.

[17] Elser JJ, Fagan WF, Denno RF, Dobberfuhl DR, Folarin A, et al (2000) Nutritional constraints in terrestrial and freshwater food webs. Nature 408: 578–580. 11117743 10.1038/35046058

[18] Ågren GI (2008) Stoichiometry and Nutrition of Plant Growth in Natural Communities. Annu Rev Ecol Evol Syst 39: 153–170.

[19] Niklas KJ, Cobb ED, Niinemets Ü, Reich PB, Sellin A, et al (2007) “Diminishing returns”. in the scaling of functional leaf traits across and within species groups.Proc Natl Acad Sci U S A 104: 8891–8896. 17502616 10.1073/pnas.0701135104 PMC1885598

[20] Yamawo A, Suzuki N, Tagawa J, Hada Y (2012) Leaf ageing promotes the shift in defence tactics in Mallotus japonicus from direct to indirect defence. J Ecol 100: 802–809.

[21] Ridley CE, Hangelbroek HH, Wagenius S, Stanton-Geddes J, Shaw RG (2011) The Effect of Plant Inbreeding and Stoichiometry on Interactions with Herbivores in Nature: Echinacea angustifolia and Its Specialist Aphid. Plos One 6: e24762. 21935460 10.1371/journal.pone.0024762 PMC3172291

[22] Yu Q, Elser JJ, He NP, Wu HH, Chen QS, et al (2011) Stoichiometric homeostasis of vascular plants in the Inner Mongolia grassland. Oecologia 166: 1–10. 21221646 10.1007/s00442-010-1902-z

[23] Hoagland DR, Arnon DI (1950) The water-culture method for growing plants without soil.Circular 347, California Agricultural Experiment Station, College of Agriculture. University of California, Berkeley.

[24] Jensen MH, Collins W (1985) Hydroponic vegetable production. Hortic Rev 7: 483–558.

[25] Kennedy J, Ibbotson A, Booth C (1950) The distribution of aphid infestation in relation to leaf age. Ann Appl Biol 37: 651–679.

[26] Walkley A, Black IA (1934) An examination of the Degtjareff method for determining soil organic matter, and a proposed modification of the chromic acid titration method. Soil Sci 34: 29–38.

[27] Bremner JM (1996) Nitrogen: total. In: D.L Sparks, A.L Page, P.A.Helmke, R.H Loeppert, P.N Soltanpour, M.A.Tabatabai, C.T Johnson & M.E Sumner, editors.Methods of Soil Analysis. Part 3: Chemical Methods.Madison: Soil Science Society of America and American Society of Agronomy.pp. 1085–1123.

[28] Kuo S (1996) Phosphorus. In: D.L Sparks, A.L Page, P.A Helmke, R.H Loeppert, P.N Soltanpour, M.A Tabatabai, C.T Johnson, M.E Sumner, editors.Methods of Soil Analysis. Part 3: Chemical Methods. Madison:Soil Science Society of America and American Society of Agronomy.pp. 869–920.

[29] Bolker BM, Brooks ME, Clark CJ, Geange SW, Poulsen JR, et al (2009) Generalized linear mixed models: a practical guide for ecology and evolution. Trends Ecol Evol 24: 127–135. 19185386 10.1016/j.tree.2008.10.008

[30] Zhang SB, Zhang JL, Slik J, Cao KF (2012) Leaf element concentrations of terrestrial plants across China are influenced by taxonomy and the environment. Glob Ecol Biogeogr 21: 809–818.

[31] R Development Core Team (2011) R: A language and environment for statistical computing. R Foundation for Statistical Computing Vienna Austria.URL http:/www.R-project.org (accessed 7 December 2011).

[32] McJannet C, Keddy P, Pick F (1995) Nitrogen and phosphorus tissue concentrations in 41 wetland plants: a comparison across habitats and functional groups. Funct Ecol 9: 231–238.

[33] Chapin III FS (1980) The mineral nutrition of wild plants. Annu Rev Ecol Evol Syst 11: 233–260.

[34] Chapin III FS, Kedrowski RA (1983) Seasonal changes in nitrogen and phosphorus fractions and autumn retranslocation in evergreen and deciduous taiga trees. Ecology 64: 376–391.

[35] Aerts R, Chapin III FS (2000) The mineral nutrition of wild plants revisited: A re-evaluation of processes and patterns. Advances in Ecological Research 30: 1–67.

[36] Reich PB, Falster DS, Ellsworth DS, Wright IJ, Westoby M, et al (2009) Controls on declining carbon balance with leaf age among 10 woody species in Australian woodland: do leaves have zero daily net carbon balances when they die? New Phytol 183: 153–166. 19383100 10.1111/j.1469-8137.2009.02824.x

[37] Kattge J, Diaz S, Lavorel S, Prentice IC, Leadley P, et al (2011) TRY–a global database of plant traits. Glob Chang Biol 17: 2905–2935.

[38] Markert B (1989) Multi-element analysis in ecosystems: basic conditions for representative sampling of plant materials. Fresenius J Anal Chem 335: 562–565.

[39] Han WX, Fang JY, Guo DL, Zhang Y (2005) Leaf nitrogen and phosphorus stoichiometry across 753 terrestrial plant species in China. New Phytol 168: 377–385. 16219077 10.1111/j.1469-8137.2005.01530.x

[40] Niklas KJ, Owens T, Reich PB, Cobb ED (2005) Nitrogen/phosphorus leaf stoichiometry and the scaling of plant growth. Ecol Lett 8: 636–642.

[41] Dijkstra FA, Pendall E, Morgan JA, Blumenthal DM, Carrillo Y, et al (2012) Climate change alters stoichiometry of phosphorus and nitrogen in a semiarid grassland. New Phytol 196: 807–815. 23005343 10.1111/j.1469-8137.2012.04349.x

[42] Cornelissen J, Lavorel S, Garnier E, Diaz S, Buchmann N, et al (2003) A handbook of protocols for standardised and easy measurement of plant functional traits worldwide. Aust J Bot 51: 335–380.

[43] McGill BJ, Enquist BJ, Weiher E, Westoby M (2006) Rebuilding community ecology from functional traits. Trends Ecol Evol 21: 178–185. 16701083 10.1016/j.tree.2006.02.002

[44] Cornwell WK, Ackerly DD (2009) Community assembly and shifts in plant trait distributions across an environmental gradient in coastal California. Ecol Monogr 79: 109–126.

[45] Bolnick DI, Amarasekare P, Araújo MS, Bürger R, Levine JM, et al (2011) Why intraspecific trait variation matters in community ecology. Trends Ecol Evol 26: 183–192. 21367482 10.1016/j.tree.2011.01.009 PMC3088364

[46] Cianciaruso M, Batalha M, Gaston KJ, Petchey O (2009) Including intraspecific variability in functional diversity. Ecology 90: 81–89. 19294915 10.1890/07-1864.1

[47] Olde Venterink H, Güsewell S (2010) Competitive interactions between two meadow grasses under nitrogen and phosphorus limitation. Funct Ecol 24: 877–886.

